# Proliferative diabetic retinopathy subtypes defined by immune defense and endothelial mitochondrial dysfunction

**DOI:** 10.1038/s41392-025-02448-9

**Published:** 2025-10-22

**Authors:** Maximilian A. McCann, Basma Baccouche, Yueru Li, Priti Roy, Neil Sheth, Jennifer I. Lim, William F. Mieler, Felix Y. Chau, Lawrence J. Ulanski, R. V. Paul Chan, Monique Munro, Robert A. Hyde, Caitlin Berek, Anna Ong, Sudeshna De, Barbara Siedlecki, Ru-Ik Chee, Yannek I. Leiderman, Michael J. Heiferman, Andrius Kazlauskas

**Affiliations:** 1 Department of Ophthalmology & Visual Sciences, University of Illinois at Chicago, Chicago, IL USA; 2 Department of Physiology & Biophysics, University of Illinois at Chicago, Chicago, IL USA

**Keywords:** Target identification, Gene expression analysis

## Abstract

Proliferative diabetic retinopathy (PDR) is a major complication of diabetes characterized by pathological angiogenesis in the retina. Standard treatment includes vitrectomy to remove these abnormal vessels, and the resulting clinical specimens provide an opportunity to define drivers of PDR. Here, we profiled endothelial and immune cells from such samples to identify disease mechanisms. In some patients, endothelial cells were more abundant, whereas in others, immune cells predominated. Immune cells exhibited gene expression programs directed against pathological endothelium, suggesting an endogenous defense that may explain the scarcity of endothelial cells in certain cases. Preoperative anti-vascular endothelial growth factor (VEGF) therapy altered transcriptional programs in both endothelial and immune cells, indicating that its effects extend beyond the vasculature. A comparison of endothelial signatures from PDR patients and nondiabetic donor retinas revealed a distinct molecular program in PDR, prominently marked by mitochondrial dysfunction. In contrast, endothelial cells from the murine oxygen-induced retinopathy (OIR) model lacked mitochondrial dysfunction, although other features of pathological angiogenesis were shared. These findings suggest that PDR is not a uniform disease but comprises distinct types characterized by either immune-mediated clearance of pathological vessels or endothelial mitochondrial dysfunction. They also revealed that anti-VEGF therapy influences both endothelial and immune compartments, with implications for treatment strategies. Finally, the data clarify both the relevance and limitations of the OIR model for preclinical testing of new therapeutic targets.

## Introduction

Diabetic retinopathy (DR) is a microvascular complication of diabetes mellitus (DM) that affects more than one in four patients with DM.^1^ There are several stages in the progression of DR. The first stage is an extended retinopathy-free period during which the retina remains healthy in the face of chronic hyperglycemia.^2^ This period of resilience to DR (RDR) delays the onset of DR for many years. When protective mechanisms are exhausted, nonproliferative DR (NPDR) develops. NPDR is characterized by mild vascular damage, including microaneurysms, capillary basement membrane thickening, and retinal hemorrhages.^3^ The progression of vascular dysfunction correlates with both the duration and severity of DM^4^, advancing to sight-threatening stages, most commonly diabetic macular edema (DME) and proliferative DR (PDR).^4^ DME is characterized by swelling of the central retina caused by increased vascular permeability, which can occur at any stage of DR.^5^ The growth of pathological blood vessels on the surface of the retina or into the vitreous is a quintessential feature of PDR.^6^ This neovascularization produces vessels which are fragile and prone to recurrent hemorrhage. The clinical features of end-stage PDR include nonclearing vitreous hemorrhage, tractional retinal detachment, and severe fibrovascular proliferation resulting in the formation of a fibrovascular membrane (FVM). PDR and DME are not mutually exclusive; patients can have both pathologies.

Vascular endothelial growth factor (VEGF) plays a central role in DR, as evidenced by the ability of VEGF-neutralizing therapies to improve vision and/or prevent further deterioration.^7–10^ Anti-VEGF agents, which are injected into the vitreous, are used in two ways to manage DR. The most common approach is to improve vision by limiting vascular leakage or the growth of pathological vessels.^7^ The second is as an adjuvant to surgery in patients with end-stage PDR. Treatment of patients with end-stage PDR involves vitrectomy surgery to remove FVMs, which exert traction on the retina.^3^ Vitrectomies also serve to reattach the retina and remove blood from hemorrhaged blood vessels.^11^ Anti-VEGF is used preoperatively in this context because it reduces VEGF and consequently reduces the risk of intraoperative bleeding^12^ and early postoperative vitreous hemorrhages.^13–15^ Anti-VEGF therapy is not used to improve vision in this context directly but rather to reduce the risk of intra- and postoperative complications. Overall, anti-VEGF therapy has transformed DR management, but still possesses limitations. A substantial subset of patients show incomplete or transient responses, while the frequency of injections constitute a high patient burden.^16^ Anti-VEGF does not reverse the underlying pathology, nor does it halt disease progression in all patients.^17^ These limitations highlight the need for alternative or complementary therapeutic strategies that address mechanisms beyond VEGF signaling, including metabolic and immune pathways.

In addition to VEGF, the immune system contributes to vascular dysfunction, as evidenced by the effectiveness of corticosteroids as a therapy for DR.^18^ Corticosteroids act by broadly suppressing pro-inflammatory signaling pathways and transcription of inflammatory mediators like cytokines and adhesion molecules that are upregulated in the diabetic retina.^19^ An investigation of the effects of corticosteroids in a murine neovascularization model demonstrated a cell type-specific influence.^20^ It is known that macrophages regulate retinal neovascularization. Macrophage polarization (M1 vs. M2) can determine whether such regulation promotes or limits neovascularization.^21–25^ The polarization state of macrophages may determine whether angiogenesis is exacerbated or suppressed during DR. Other cell types (neutrophils and monocytes) act on pathological blood vessels to drive their regression.^20,26,27^ Examination of PDR membranes demonstrates the presence of diverse immune infiltrates including macrophages, neutrophils, and lymphocytes, supporting the concept that immune-vascular interactions contribute to disease progression.^28,29^ Collectively, these findings establish that DR also features an immune component. The ability of the immune system to modulate neovascularization points to opportunities to develop new PDR therapeutics outside of the VEGF arena.

Clinical specimens from patients with end-stage PDR constitute an opportunity to identify drivers of pathological angiogenesis in patients. Previous studies have analyzed FVMs, which are relatively easy to isolate during vitrectomy surgery. These studies defined the cellular composition of FVMs^28,30^ and reported the gene expression profiles of this heterogeneous cell mixture.^31,32^ Analysis of endothelial cells within FVMs^28,30^ indicated that they expressed markers of both blood and lymphatic endothelium.^28,32^ While these studies revealed the gene expression profile of the endothelium residing in the unique microenvironment of the FVM, the PDR signature of endothelial cells in pathological blood vessels outside of the FVM (within the vitreous) remains unexplored. In this study, we profiled the molecular landscape of endothelial and immune cells within the vitreous of patients with end-stage PDR. These findings indicate that PDR is a heterogeneous disease comprising distinct forms driven either by immune-mediated clearance of pathological vessels or by endothelial mitochondrial dysfunction. They further show that anti-VEGF therapy impacts both endothelial and immune compartments, underscoring implications for treatment. In addition, the results refine our understanding of the oxygen-induced retinopathy model, highlighting both its utility and its limitations for evaluating new therapeutic targets in preclinical studies.

## Results

### Isolation of CD31^+^ cells from the vitreous of patients with end-stage PDR

**Fig. 1:**
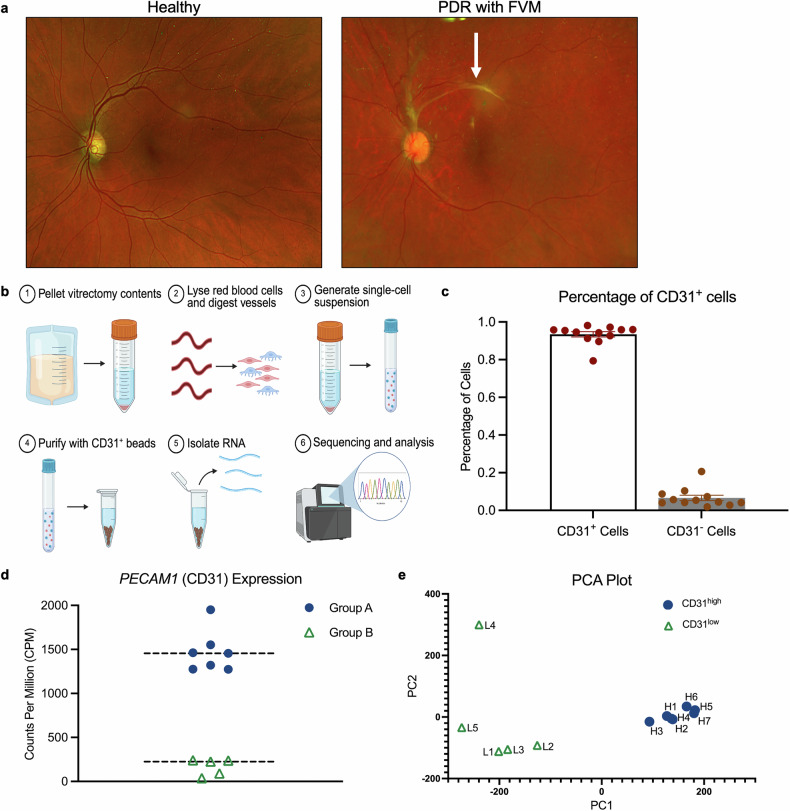
CD31^+^ cells in the vitreous of patients with PDR are not all endothelial cells. **a** Fundus image depicting a patient with a healthy retina (left) and the vasculature and fibrovascular membrane of a patient with PDR (right). A fibrovascular membrane (arrow) is present overlying the retinal blood vessels, causing localized tractional retinal detachment in the superior macula. End-stage PDR eyes exhibit vitreous hemorrhage, which prevents a clear view of the pathological features that are present on the surface of the retina. **b** A diagram depicting the procedure for isolating CD31^+^ cells from the vitrectomy cassette obtained from patients with end-stage PDR. **c** Bar graph depicting the percentage of CD31^+^ cells present in each sample. The cell type proportion was estimated via CIBERSORT, after which the proportions of all cell types that expressed *PECAM1* (the gene encoding CD31) in the peripheral retina reference dataset were combined. The data are presented as the means ± SDs, with each point representing a gene expression signature. **d** Scatter plot depicting the counts per million *PECAM1* gene in the gene expression signatures of CD31^+^ cells isolated from vitrectomy cassettes. The mean values for each group are presented with dashed lines. **e** Principal component analysis (PCA) scores of the CD31^high^ and CD31^low^ gene expression signatures. The first two principal components are shown

The pathological blood vessels that develop in patients with PDR are within the vitreous, just above the retina (Fig. 1a). These blood vessels are fragile and can bleed, resulting in vitreous hemorrhage. Over time, these blood vessels grow on a scaffold of FVMs that pull on the underlying retina, causing tractional retinal detachment. Both vitreous hemorrhage and tractional retinal detachment are common causes of vision loss in patients with PDR and often require surgery to improve or stabilize the patient’s vision.

Pars plana vitrectomy is a surgical procedure that removes the vitreous humor and the scaffold upon which new vessels can grow.^33^ The pathological blood vessels that develop in patients with PDR are removed along with the vitreous during pars plana vitrectomy. The vitreous can be removed to treat vision loss related to vitreous hemorrhage. Removing pathological blood vessels relieves the traction that these vessels are exerting on the retina and allows the retina to reattach after surgery, which usually stabilizes retinal damage and can improve vision.

Surgically removed vitreous fluid was processed to isolate CD31^+^ cells as outlined in the Materials and Methods section and illustrated in Fig. 1b. The RNA from CD31^+^ cells from 13 patients was subjected to bulk RNA-Seq. The depth of sequencing was approximately 57 million average reads per sample for 12/13 samples; one sample had a reduced sequencing depth (10 million reads) and was excluded from subsequent analysis. The reads of the remaining 12 samples were trimmed, and 85.2 ± 10.2% of the trimmed reads were mapped to the genome.

### The CD31^+^ cells in the vitreous of patients with end-stage PDR are not only endothelial

Analysis of the resulting gene expression data indicated that more than 90% of the cells that were sequenced were CD31-expressing cells (Fig. 1c). Curiously, the level of *PECAM1* (the gene encoding CD31) expression segregated the 12 patients into two groups: A and B, whose cells expressed high and low levels of *PECAM1*, respectively (Fig. 1d). Comparison of global gene expression in these cells revealed that many genes were expressed similarly within a group and differently between the two groups (Fig. 1e). The discovery that patients with end-stage PDR could be stratified on the basis of the gene expression profile of the cells in their vitreous is a novel and surprising observation. The clinical features of these CD31^high^ and CD31^low^ patient groups were indistinguishable (Table 1).

**Table 1:** Clinical parameters of the patients

Parameter	CD31^high^ (*n* = 7)	CD31^low^ (*n* = 5)
Age	50 ± 10.6	49.8 ± 18.9
HbA1c	8.64 ± 1.5	9.76 ± 3.1
Sex (Male/Female)	3/4 (43/57%)	3/2 (60/40%)
Type 1 DM	1 (14.3%)	3 (60%)
Type 2 DM	6 (85.7%)	2 (40%)
Retinal Detachment (RD)	4 (57.1%)	1 (20%)
Fibrovascular Proliferation (FVP)	3 (42.9%)	2 (40%)
RD or FVP	6 (85.7%)	2 (40%)
Diabetic Macular Edema	3 (42.9%)	4 (80%)
Retinal Tear	3 (42.9%)	0 (0%)
Preop. anti-VEGF ( < 1 month)	3 (42.9%)	2 (40%)
Systemic Prednisolone/Steroids	1 (14.3%)	2 (40%)
PRP or EndoLaser	7 (100%)	5 (100%)
Prior surgery in sampled eye	0 (0%)	2 (40%)
CEIOL or SFIOL	4 (57.1%)	1 (20%)
360 retinectomy	1 (14.3%)	0 (0%)
Tamponade used at end of surgery	4 (57.1%)	2 (40%)
Lens Status (Phakic/Pseudophakic)	7/0 (100/0%)	3/2 (60/40%)

Clinical parameters of patients in group A and group B. Data are presented as the means ± SDs or as the number of patients and percentages (%)

*PRP* panretinal photocoagulation, *CEIOL* cataract extraction and intraocular lens insertion, *SFIOL* scleral fixated intraocular lens

**Fig. 2:**
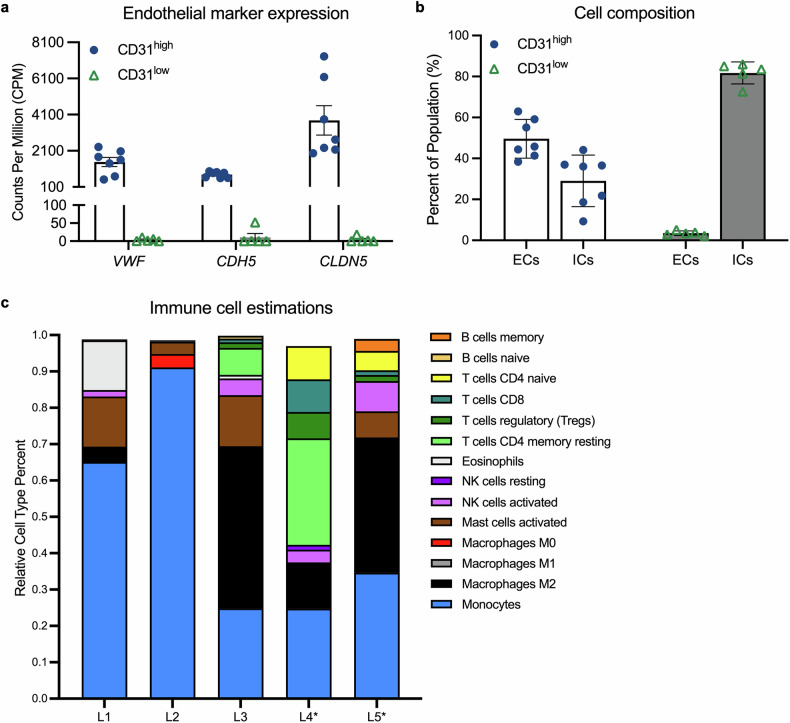
CD31^+^ cells are either endothelial or immune cells. **a** Bar graph depicting the counts per million of the endothelial marker genes *VWF*, *CDH5*, and *CLDN5* in the CD31^high^ and CD31^low^ gene expression signatures. The data are presented as the means ± SDs, with each point representing a gene expression signature of an individual patient. **b** Bar graph depicting the percentage of endothelial (ECs) and immune (ICs) cells present within CD31^+^ populations isolated from vitrectomy cassettes in the CD31^high^ and CD31^low^ groups. The data are presented as the means ± SDs, with each point representing a gene expression signature. **c** The cell type proportions were estimated via CIBERSORT. Stacked bar chart depicting the estimated proportions of different immune cell types within the CD31^low^ populations (L1–L5; shown in Fig. 1e) isolated from the vitreous. The 14 immune cell types with the highest estimations are shown. Asterisks* denote patients who received preoperative anti-VEGF

We proceeded to identify the cell types that were present in these two groups of patients. The cells from the CD31^high^ patients expressed high levels of additional endothelial markers, whereas those from the CD31^low^ patients did not (Fig. 2a). Furthermore, CIBERSORT-based analysis revealed that the major cell type in CD31^high^ patients was endothelial cells (Fig. 2a). In contrast, the major cell type in CD31^low^ patients was immune cells (Fig. 2b), which included monocytes (48.1 ± 29.2%), macrophages (20.5 ± 19.2%), T cells (15.1 ± 22.6%), and mast cells (8.2 ± 5.5%) (Fig. 2c). These data align with previous publications reporting that CD31 is not an endothelial-specific marker. CD31 is also expressed on various immune cells, including neutrophils, monocytes, lymphocytes, and natural killer cells.^34^ Thus, the recovery of endothelial and immune cells was not surprising because both express CD31. While we anticipated that endothelial cells would be more abundant than immune cells, as is the case for most Group A/CD31^high^ patients (Fig. 2b), we did not expect that immune cells would be the predominant cell type in some of the patients (Group B/CD31^low^) (Fig. 2b).

### Immune cells in CD31^low^ patients display a gene signature associated with the elimination of pathological blood vessels

**Fig. 3:**
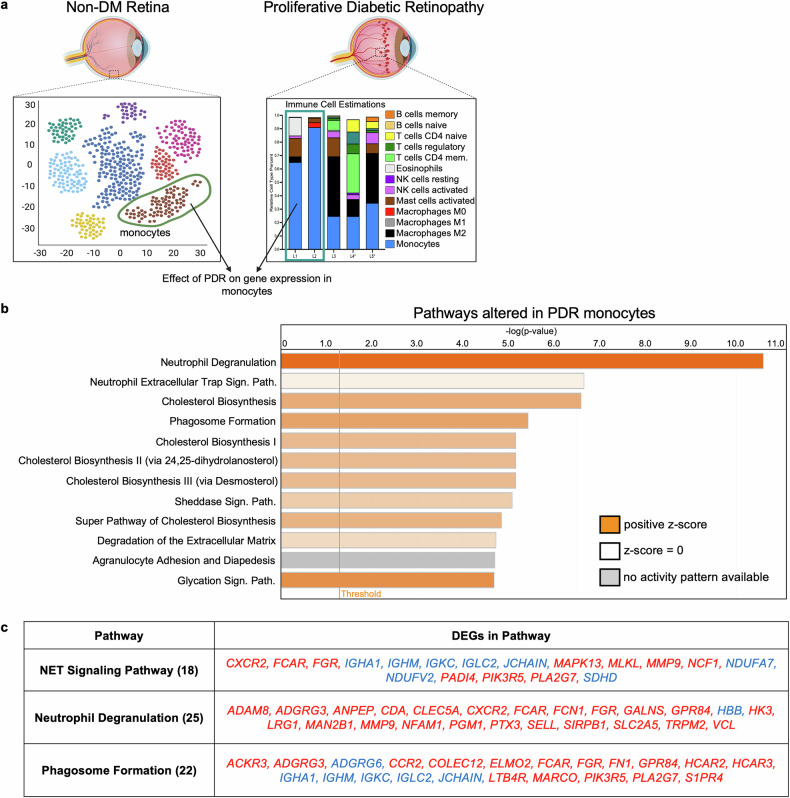
Immune cells within the vitreous of patients with end-stage PDR have a gene signature associated with vascular repair. **a** Schematic depicting the strategy used to establish the molecular signature of PDR in monocytes. Relative expression ordering analysis (REOA) was used to compare the gene expression signatures of monocytes from non-DM retinas to the monocyte-enriched PDR signatures. **b** List of the 12 pathways most significantly altered in the PDR monocyte signature, as determined by Ingenuity Pathway Analysis (IPA). IPA was performed on the 289 differentially expressed genes that presented the greatest expression changes in the PDR monocytes. Pathways are sorted by statistical significance, with the bar color depicting the pathway activity z score. **c** Table depicting the differentially expressed genes altered in the immune clearance-associated pathways in the PDR monocyte signature, as determined by IPA. Genes in red text were upregulated in PDR monocytes, and genes in blue text were downregulated

We compared the gene expression signatures of CD31^low^ patients, which primarily contained monocytes (L1 and L2 in Fig. 2c), with the expression signatures of monocytes from non-DM/DR donors (Fig. 3a, Data S1).^35^ This analysis revealed that immune cells in CD31^low^ patients were programmed to eliminate pathological blood vessels. The monocytes of these patients presented upregulated pathways (neutrophil degranulation, extracellular trap signaling, and phagosome formation) that eliminate pathological blood vessels (Fig. 3b). In the other CD31^low^ patients, M2 macrophages were a prominent cell type, while M1 polarization was absent. We speculate that CD31^low^ patients have few endothelial cells because they are cleared by immune cells. If this is indeed the case, then it suggests that immune-based defense against pathological blood vessels operates even in the most advanced stages of PDR.

Further analysis of the differentially expressed genes provided additional mechanistic insight into this phenomenon. *PADI4*, which encodes PAD4, a key enzyme in extracellular trap formation, was enriched in PDR monocytes (Fig. 3c). Deletion of PAD4 in LysM-expressing cells slows the regression of pathological vessels in a mouse model of neovascularization.^26^ Similarly, *CXCR2* and *CCR2* (which encode receptors for CXCL1 and MCP1, respectively) were also enriched in PDR monocytes (Fig. 3c). In experimental animals, these receptors and their corresponding ligands are key to the formation of extracellular traps and the resolution of pathological blood vessels.^26,27^ Taken together, these gene expression changes suggest that PDR monocytes are recruited to pathological vessels to eliminate them.

### Anti-VEGF agents alter gene expression within immune cells

**Fig. 4:**
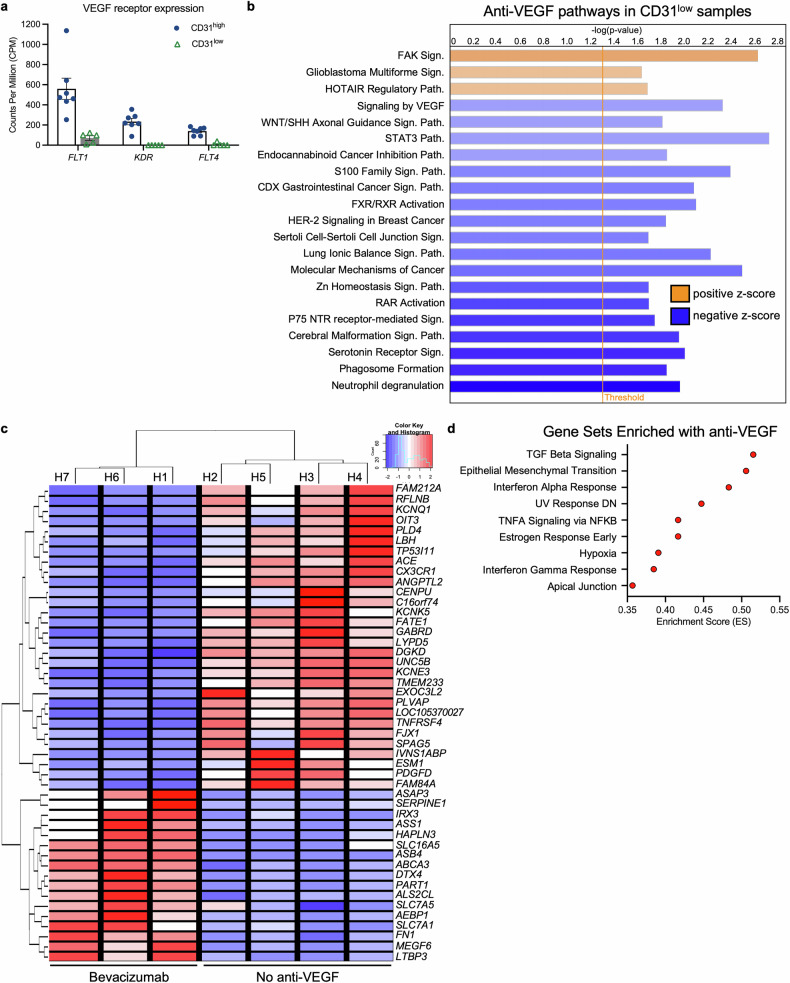
Anti-VEGF agents altered gene expression in immune and endothelial cells. **a** Bar graph depicting the counts per million of the VEGF receptor genes *FLT1*, *KDR*, and *FLT4* in the CD31^high^ and CD31^low^ gene expression signatures. The data are presented as the means ± SDs, with each point representing a gene expression signature. **b** List of the pathways with the strongest changes in activity in the CD31^low^ populations from patients who received preoperative aflibercept, as determined by IPA. Pathways are sorted by z score, with orange or blue depicting a positive or negative activity z score, respectively. **c** Heatmap displaying the genes that are differentially expressed (FDR < 0.05) in the endothelium of patients who received preoperative bevacizumab. Z scores were calculated across all samples for each gene and mapped along a color gradient with negative z scores in blue, zero in white, and positive z scores in red. Each column is labeled with the sample ID within the CD31^high^ (H1–H7) group. *n* = 3–4 patients per group. **d** Dot plot depicting the gene sets enriched in the gene signature of CD31^high^ patients who received preoperative bevacizumab, as determined by gene set enrichment analysis (GSEA). Gene sets are organized by enrichment score

Because nonendothelial cells express VEGF receptors and respond to VEGF,^36^ we investigated whether the effect of anti-VEGF extends beyond the vasculature. This possibility is relevant in the context of patients with end-stage PDR because cells from CD31^low^ patients expressed VEGFR1 (*FLT1*, Fig. 4a). VEGFR1 expression levels differed between CD31^high^ and CD31^low^ patients, potentially due to differences in cell type (Fig. 2), which is a key determinant of VEGFR1 expression.^37^ We proceeded to investigate the effect of anti-VEGF therapy on these VEGFR1^+^ nonendothelial cells by comparing the gene expression profiles of CD31^low^ patients who had and had not been treated preoperatively with anti-VEGF therapy. The expression of 178 genes was affected by anti-VEGF agents (Supplementary Fig. 1, Data S2), which suggests that nonendothelial cells in the vitreous of patients with end-stage PDR respond to VEGF and are therefore affected by anti-VEGF agents. Pathway analysis supported this concept because the “signaling by VEGF” pathway was suppressed in CD31^low^ patients who received preoperative anti-VEGF (Fig. 4b). Furthermore, anti-VEGF therapy suppressed inflammation. The “S100 family signaling pathway”, which acts via NFKB to promote inflammation,^38^ and the “STAT3 pathway”, which is engaged by inflammatory cytokines, were suppressed (Fig. 4b). The “neutrophil degranulation” and “phagosome formation” pathways were also downregulated. Together, these data reveal that anti-VEGF agents alter gene expression in nonendothelial cells and that such changes may contribute to the beneficial effects of anti-VEGF agents.

We also investigated the effect of systemic steroids on gene expression in nonendothelial cells by comparing the gene expression profiles of CD31^low^ patients who did and did not receive systemic prednisolone. The expression of five genes was altered in patients who received prednisolone (Supplementary Fig. 2a). While there are confounding factors that affect gene expression, such as cell type composition or treatment with anti-VEGF agents (Figs. 2c and 4b), there were stark differences in the expression of these genes between patients who did and did not receive prednisolone. These changes included the fatty acid transporter CD36, which is suppressed by the glucocorticoid dexamethasone (Supplementary Fig. 2b).^39^ Since CD36 contributes to macrophage polarization,^40^ prednisolone may affect the polarization of these cells within the eyes of these patients. Versican, a matrix proteoglycan encoded by *VCAN*, is known to bind and retain myeloid and lymphoid cells within a tissue.^41^ Its reduced expression could affect the retention of immune cells within the lesion. Finally, the expression of *TNFRSF10B* (encodes for death receptor 5) was also reduced, which could have prosurvival effects on nonendothelial cells exposed to prednisolone. In summary, prednisolone affects gene expression within nonendothelial cells and can impact the inflammatory response.

### Anti-VEGF-regulated genes—potential alternatives to anti-VEGF

We also identified anti-VEGF-regulated genes in CD31^high^ patients, in whom endothelial cells were the major cell type (Fig. 2b). A pairwise comparison of gene expression between CD31^high^ patients who did and did not receive preoperative anti-VEGF therapy revealed 47 differentially expressed genes (DEGs) (Fig. 4c). This list included canonical VEGF-regulated genes (*PLVAP*, *ACE*, *UNC5B*, *ESM1*, *KCNE3*, and *EXOC3L2*)^42–45^ and thereby demonstrated concordance between in vitro models consisting of cultured endothelial cells and pathological blood vessels in patients. Anti-VEGF agents also regulate the expression of genes that are not known to be downstream of VEGF. The known functions of the proteins encoded by some of these genes (transporters for ions (*KCNQ1*), phospholipids (*ABCA3*), metabolites and amino acids (*SLC16A5* and *SLC7A1*)) highlight our incomplete understanding of how VEGF/anti-VEGFs govern vascular homeostasis.

To further analyze the anti-VEGF-regulated genes, we subjected them to gene set enrichment analysis (GSEA). The results identified gene sets that are likely to mediate the beneficial effects of anti-VEGF agents. Anti-VEGF increased the expression of the “Hallmark apical junction” gene set (Fig. 4d), which includes genes encoding junctional proteins that govern blood vessel permeability, namely, adherens junctions (cadherins) and tight junctions (claudins, TJPs). Similarly, gene sets associated with proliferation (“Hallmark E2F targets”, “Hallmark G2M checkpoint”, and “Hallmark mitotic spindle”), which are intrinsic to angiogenesis, were reduced in anti-VEGF-treated patients (Supplementary Fig. 3). Thus, anti-VEGF agents change gene expression in ways that curb the leakage and angiogenesis of blood vessels.

Further interrogation of this dataset provided a molecular explanation for anti-VEGF-mediated fibrosis.^46,47^ The “Hallmark TGFb signaling” and “Hallmark epithelial mesenchymal transition” gene sets were enriched in patients treated with anti-VEGF (Fig. 4d). These data indicate that some of the detrimental effects of anti-VEGF agents are likely to be mediated by anti-VEGF-driven changes in gene expression. In contrast, no profibrotic factors were altered in the CD31^low^ samples subjected to preoperative anti-VEGF therapy (Data S2). Thus, it appears that the detrimental, profibrotic effect of anti-VEGF manifests within endothelial cells instead of immune cells.

### Mitochondrial dysfunction is a prominent feature of the PDR signature within the endothelium

**Fig. 5:**
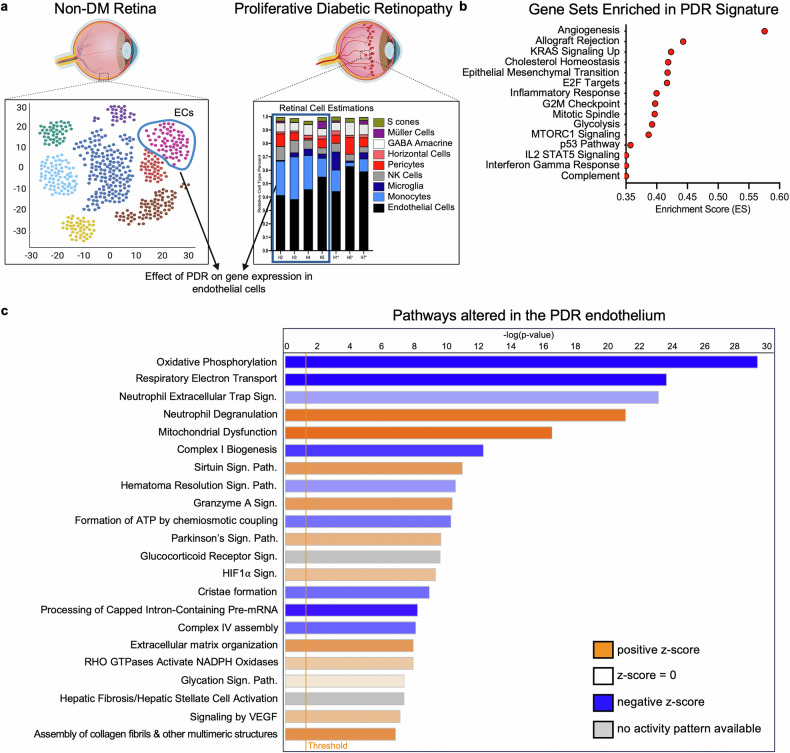
The molecular signature of PDR is associated with mitochondrial dysfunction. **a** Schematic depicting the strategy used to establish the molecular signature of PDR in the endothelium. Relative expression ordering analysis (REOA) was used to compare the gene expression signatures of endothelial cells from non-DM retinas to the endothelial cell-enriched PDR signatures. The PDR signatures of CD31^high^ patients (*n* = 4) and three previously sequenced endothelial cell-enriched samples who did not receive preoperative anti-VEGF therapy were used for this comparison. **b** Dot plot depicting the gene sets enriched in the PDR signature, as determined by gene set enrichment analysis (GSEA). Gene sets are organized by enrichment score. **c** List of the pathways most significantly altered in the signature of the PDR endothelium, as determined by Ingenuity Pathway Analysis (IPA). IPA was performed on the genes differentially expressed within the PDR endothelium. Pathways are sorted by statistical significance, with the bar color depicting the pathway activity z score

We defined the molecular signature of PDR within the endothelium by comparing the endothelial-enriched signatures of CD31^high^ patients who did not receive anti-VEGF therapy with the transcriptomes of non-DM retinal endothelial cells (Fig. 5a). This strategy identified 856 genes that were differentially expressed in the PDR endothelium (315 upregulated, 541 downregulated) (Data S3). These genes constitute the PDR signature of treatment-naïve CD31^high^ patients.

Pathway analysis of the PDR signature confirmed both established and some of the emerging concepts related to PDR pathogenesis. The “hallmark angiogenesis” gene set and the “signaling by VEGF” pathway were enriched within the PDR signature (Fig. 5b, c).^48–50^ Similarly, the PDR signature contained evidence of mitochondrial dysfunction (enrichment of the “mitochondrial dysfunction” pathway and suppression of the “oxidative phosphorylation” and “respiratory electron transport” pathways (Fig. 5c)), which is known to occur within the diabetic retina.^51–53^ The emerging concepts that were detected in the PDR signature included endothelial senescence (“hallmark p53 signaling” and “hallmark interferon gamma response” gene sets) (Fig. 5b).^54–57^ Finally, the PDR signature provides novel concepts regarding PDR pathogenesis. The enrichment of pathways affecting the organization of the extracellular matrix (ECM) (“ECM Organization” and “Assembly of Collagen Fibrils & Other Multimeric Structures” pathways (Fig. 5c)) suggests that the endothelium contributes to the pathological microenvironment of the vasculature in the context of clinical PDR.

In summary, the PDR-associated gene expression changes that occur within the endothelium include established (VEGF signaling, mitochondrial dysfunction), emerging (senescence), and novel (ECM organization) concepts related to the pathogenesis of PDR.

### Some of the PDR signature is present in the mouse model of OIR

**Fig. 6:**
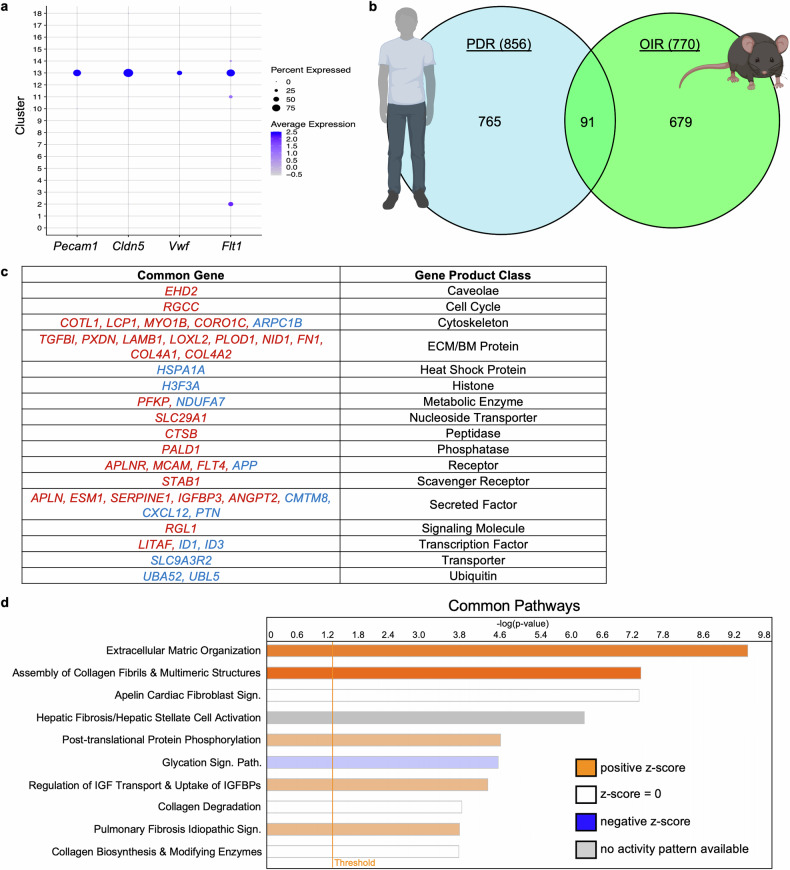
Matrix reorganization was one of the strongest components of both the PDR and OIR signatures. **a** Dot plot depicting the frequency and expression of the endothelial markers *Pecam1*, *Cldn5*, *Vwf*, and *Flt1* within the cell clusters obtained from normoxic and OIR mouse retinas. The dot size corresponds to the percentage of cells within the clusters that express each marker, and the shade of blue corresponds to the average expression within the clusters. **b** Venn diagram depicting the number of genes that are common between the PDR and OIR signatures. **c** Table depicting the genes that are commonly regulated in the PDR and OIR expression signatures, along with the functions of their gene products. **d** List of the 10 pathways most significantly altered in the genes commonly regulated in the PDR and OIR endothelia, as determined by Ingenuity Pathway Analysis. Pathways are sorted by statistical significance, with the bar color depicting the pathway activity z score

Next, we compared the molecular signatures within the endothelium of pathological blood vessels that develop in patients with PDR and in an oxygen-induced retinopathy (OIR) mouse model. To obtain the OIR signature within retinal endothelial cells, we reanalyzed a publicly available single-cell RNA-Seq (scRNA-Seq) database from OIR retinas (Fig. 6a, Supplementary Fig. 4).^26^ As expected, this signature included increased expression of angiogenesis-related genes such as *Hif1a, Nrp1*, *Nrp2*, *Esm1*, *Kdr*, *Notch4*, and *Angpt2* (Data S4). A comparison of the OIR and PDR signatures revealed 91 common genes, 43 of which were regulated in the same direction in both signatures (Fig. 6b). Many of these genes have been previously reported in angiogenic settings (Fig. 6c). This list includes *ANGPT2*, which is a target for FDA-approved therapies to treat patients with DR (DME, but not PDR).^58^

Subsequent analysis of the common genes identified components of the PDR signature that were reflected in the mouse model. These included pathways that affect the level and organization of the ECM (“ECM organization” and “assembly of collagen fibrils & other chimeric structures” pathways) (Fig. 6d). The presence of the “hepatic fibrosis”, “collagen degradation”, “collagen biosynthesis”, and “pulmonary fibrosis” pathways among the top ten pathways further supports the idea that ECM remodeling is a component of PDR pathogenesis that can be investigated in an OIR mouse model.

### Dissonance between the murine OIR model and clinical PDR

**Fig. 7:**
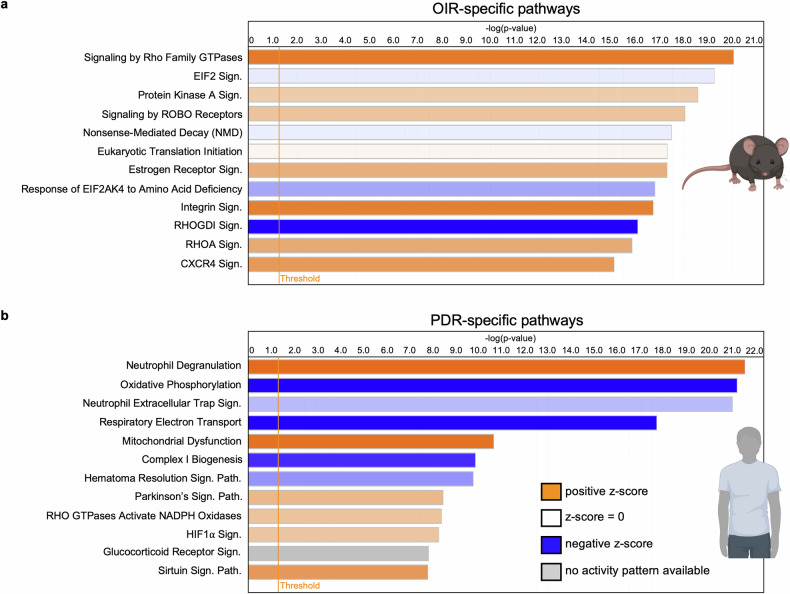
The OIR signature does not include the mitochondrial dysfunction observed in the PDR signature. List of the 12 pathways most significantly altered in the genes specific to the OIR signature (**a**) or PDR signature (**b**) as determined by IPA. Pathways are sorted by statistical significance, with the bar color depicting the pathway activity z score

Subsequent comparisons of the PDR and OIR signatures focused on differences in the molecular landscape of endothelial cells in pathological blood vessels of patients with end-stage PDR and the OIR model (Fig. 7). While in both scenarios, retinal ischemia is the driver of pathological angiogenesis, the cause of ischemia (varying the oxygen content of the air that animals breathe versus diabetes-induced capillary degeneration) and the nature of the vasculature (immature and undergoing development, versus mature undergoing prolonged metabolic stress) are different and provide the basis for differences in the molecular landscape of the endothelium. Indeed, there were unique components of each of the signatures. Only the OIR signature included development-related changes (e.g., activation of the “signaling by ROBO receptors” pathway) (Fig. 7a),^59^ whereas only the PDR signature harbored metabolism-related perturbations (e.g., activation of the “mitochondrial dysfunction” pathway) (Fig. 7b). These data identify components of the mouse OIR model that do and do not reflect the pathogenesis of patients with PDR. Focusing on the components that reflect patient pathology (Fig. 6) will improve the success of efforts using the OIR model to develop new therapeutic approaches and biomarkers.

## Discussion

The development of the next generation of PDR therapeutics will require an advancement in our current understanding of the vascular pathology that defines the disease and identification of the molecular mediators responsible for the clinical benefits of the current therapeutics (anti-VEGFs). In this study, we defined the molecular signature of PDR in the endothelium of patients, identified alternative targets to the frontline target (VEGF), and delineated components of pathological angiogenesis that can be utilized in a murine preclinical model.

The findings described herein indicate that patients with PDR do not all have the same flavor of PDR. In some patients, the immune system resolves pathological blood vessels. Unfortunately, the success of this endeavor does not restore vision because of additional issues, such as fibrotic membranes causing retinal detachment. In other patients, pathological blood vessels are present, and the molecular landscape within the endothelium reflects both traditional (mitochondrial dysfunction) and novel (ECM remodeling) features of vascular dysfunction within the retina. The determinants of a given type of PDR and whether they are mutually exclusive are yet to be determined.

The results presented herein suggest the existence of an immune-based defense against pathological angiogenesis. While spontaneous regression of neovessels has been observed in a small number of PDR patients,^60,61^ the cause of such regression has not been established. We found that the monocytes within the vitreous PDR of CD31^low^ patients harbored a gene expression signature indicating their ability to clear pathological vessels (Fig. 3b). The near absence of endothelial cells in the vitreous of CD31^low^ patients supports this concept. However, additional analysis of the PDR signature in monocytes indicated that they also upregulated pathways that may contribute to pathogenesis, such as increased cholesterol synthesis, which can lead to the formation of pathology-inducing crystals.^62,63^ Thus, the contribution of these immune cells to PDR may be complex. The increasing awareness of the role of the immune system in PDR warrants re-evaluation of the use of broadly acting immunosuppressants such as corticosteroids.^64^

Immune-mediated resolution of pathological angiogenesis also occurs in the OIR animal model and reflects at least some of what occurs in patients. Macrophages infiltrate neovascular tufts in an MCP-1/CCL2-dependent manner, where they promote apoptosis-driven regression of pathological blood vessels.^27,65–67^ Similarly, senescent endothelial cells within pathological blood vessels recruit neutrophils and thereby facilitate their clearance.^26^ However, other types of immune cells (e.g., CX3CR1-positive cells) promote pathological angiogenesis in OIR models.^20^ Curiously, these cells are pathogenic only after pathological angiogenesis has commenced.^20^ The growing realization that immune-mediated resolution of pathological blood vessels occurs in patients^26^ increases the ways in which the OIR model reflects clinical PDR: not only ischemia-induced angiogenesis but also immune-mediated resolution of pathological blood vessels.

The results presented herein align with the growing appreciation for the role of VEGF outside of the vasculature. In the context of cancer, neutralizing VEGF not only curbs tumor angiogenesis but also rewires the immune system in ways that increase its antitumor ability.^36,68^ Our observation that anti-VEGF suppressed inflammation-related pathways in PDR immune cells indicates that, in the context of clinical PDR, VEGF acts on the immune system to promote inflammation.

We were unable to identify clinical features that distinguish CD31^high^ patients from CD31^low^ patients (Table 1). Similarly, the postoperative fundus and OCT images were comparable with respect to macular edema, retinal detachment, and retinal atrophy (data not shown). Preoperative fundus photos were not taken since the vitreous hemorrhage precluded a clear view of the fundus. Clinical features that are not available and will be considered in future attempts to distinguish CD31^high^ and CD31^low^ patients include the initial intraoperative perfusion status of the FVP; the configuration of the TRD, whether the entire macula was detached from the TRD or just a few focal points; and the posterior hyaloid status, whether it was completely attached, partially elevated, or completely detached. Similarly, the chronicity of the TRD may contribute to the difference between the CD31^high^ and CD31^low^ groups; some patients have good vision and then develop fast-progressing TRDs within a few weeks (e.g., more common in patients with type 1 diabetes), whereas others present with > 6-month chronic TRDs and vision loss.

The identification of molecular mediators of anti-VEGFs is the first step in developing alternatives to anti-VEGF agents that retain pro-therapeutic features while lacking features that are anti-therapeutic. One such pro-therapeutic feature is the suppression of VEGF-induced angiogenesis. Markers of tip cells (*ESM1*, *KCNE3*) were reduced in the endothelial-enriched cells from patients who received preoperative anti-VEGF (Fig. 4c). Several known proangiogenic genes were also downregulated in patients who received anti-VEGF therapy. *PDGFD* and *ANGPTL2*, which encode proangiogenic and vasculogenic factors, respectively, were both downregulated in patients who received anti-VEGF.^69,70^ Conversely, proangiogenic genes (*SLC7A5*, *SERPINE1*, and *IRX3*) were upregulated in patients who received anti-VEGF,^71–74^ suggesting that anti-VEGF does not completely antagonize angiogenesis in pathological vessels. Identifying the molecular mediators of anti-VEGF will enable the development of approaches that induce only the beneficial effects of anti-VEGF.

There are several limitations to this study. The small number of patients raises the possibility that our findings are not representative of patients with end-stage PDR. Furthermore, the endothelial PDR signature was derived from an enriched (instead of a pure) population of endothelial cells. This caveat is, at least in part, mitigated by the presence of genes that govern vascular homeostasis within the PDR signature. Finally, while we have not demonstrated that the newly generated database includes viable candidates for novel PDR therapeutics, we are encouraged by the presence of angiopoietin 2 within this database because it is a target of an FDA-approved therapy for patients with ocular indications resulting from vascular dysfunction.^58^

## Materials and methods

### Study design

The goal of this study was to profile the molecular landscape of CD31^+^ cells within the vitreous of patients with end-stage PDR. The research subjects were patients with end-stage PDR; their clinical characteristics are shown in Table 1. The experimental design involved the isolation of CD31^+^ cells from vitrectomy cassettes obtained from research subjects, which were subsequently subjected to RNA sequencing (RNA-Seq) analysis. RNA-Seq data were collected and processed randomly before the establishment of groups based on the extent of endothelial enrichment. Subgroups of patients who received preoperative anti-VEGF therapy, on the basis of surgeon preference, were established for pairwise comparisons. Additionally, a rank-based approach was used to compare the gene expression of the PDR samples to that of non-DM retinal cells from a reference scRNA-Seq dataset.

### Isolation of patient cells

Cells expressing CD31 were isolated from vitrectomy cassette specimens collected from patients with end-stage proliferative diabetic retinopathy (PDR). Vitrectomy cassettes were collected following pars planar vitrectomy surgery performed as the standard of care. All patients with PDR requiring vitrectomy were considered eligible for participation in the study.

To isolate the CD31^+^ populations, the material within the vitrectomy cassette was pelleted by centrifuging conical tubes at 1000 × *g* for 5 min at 4 °C. The tubes were rotated 180° and spun again in all centrifugation steps. The red blood cells (RBCs) were then lysed in RBC buffer (150 mM ammonium chloride, 100 mM sodium bicarbonate, and 10 mM EDTA disodium) for 10 min at room temperature. The pellet was then washed in cold PBS and centrifuged. The pellet was then digested using a cocktail of type II collagenase (3000 U/mL, 100502 MP Biomedicals), DNase (600 U/mL, 18047019 Thermo Fisher Scientific), and hyaluronidase (0.3%, 02100740 MP Biomedicals) in Buffer 1 (PBS + 1% BSA) for 50 min at 37 °C. The pellet was then washed in cold PBS and centrifuged as before. The pellet was reconstituted in Buffer 1 and passed through a 35 µm cell strainer (352235 Corning Life Sciences) to generate a single-cell suspension. The single-cell suspension was then mixed with magnetic Dynabeads coated with anti-CD31 antibody (4 × 10^7^ beads, 11155D Invitrogen) and rotated for 20 min at 4 °C. The CD31^+^ cells were pulled down via a magnetic tube stand and washed three times with cold PBS. The isolated cells were then lysed with RT+ buffer (Qiagen), vortexed, and frozen for batch RNA isolation.

RNA was isolated from the entire batch of samples simultaneously. Each sample underwent further homogenization via QIAshredder columns (79656 Qiagen) before being loaded onto a gDNA eliminator column (Qiagen). RNA was then extracted from the samples via the RNeasy Plus Micro Kit (74034 Qiagen). RNA quality was assessed prior to sequencing via an RNA bioanalyzer (Agilent).

### RNA sequencing & QC

RNA from 13 samples was subjected to library preparation and sequenced via NOVASeqX (Illumina). RNA-Seq resulted in approximately 57 million average reads per sample. One sample had a reduced sequencing depth (10 million reads) and was excluded from subsequent analysis. Basic processing of the raw data was performed by the University of Illinois at Chicago Research Informatics Core, as described previously.^75^ General quality control was performed via FastQC,^76^ after which the reads were trimmed via Cutadapt.^77^ The trimmed reads were aligned to the v.hg38 human reference genome in a splice-aware manner via STAR.^78^ Among the trimmed reads, 85.2 ± 10.2% mapped to the genome. The feature (gene) counts were quantified on the basis of read alignments via featureCounts.^79^ Gene isoforms were quantified via Kallisto, which uses k-mer-based pseudoalignment and expectation maximization to assign reads to isoforms probabilistically.^80^ Principle component (PC) analysis was performed on the normalized counts via the edgeR software package (v4.2.2).^81,82^

### CIBERSORT analyses

The cell type proportions were estimated for the CD31^+^ cells isolated from vitrectomy cassettes via cell type identification via estimation of relative subsets of RNA transcripts (CIBERSORT). The normalized gene expression count and associated cell type data of peripheral retinal cells were collected from the reference dataset.^35^ These data were then used to build a custom signature matrix for use with the CIBERSORTx website (https://cibersortx.stanford.edu/)^83^ via the following parameters: k.max: 999; q.value: 0.01; G.min: 300; and G.max: 500. To perform ‘cell fraction’ analysis, which involves the proportions of different cell populations in bulk tissue samples profiled by RNA-Seq, our query raw RNA-seq count data were also normalized to reference data, and the same genes were retained to prepare a “mixture”. The cell fraction was run via 1000 permutations and in relative mode. To estimate the proportions of immune cells in group B samples, the cell fraction was enumerated via the LM22 signature matrix and was run via 1000 permutations in relative mode.^84^

### Determination of anti-VEGF DEGs

To determine the anti-VEGF differentially expressed genes (DEGs), differential statistics were performed on the normalized feature counts via the edgeR software package. DEGs were identified as genes with a false discovery rate (FDR) lower than 5% (0.05) between the gene expression signatures of patients treated with or without anti-VEGF therapy during the preoperative period. The FDR was determined via the Benjamini‒Hochberg procedure.^85^ DEGs were similarly determined for CD31^low^ patients treated with the systemic prednisolone. The CD31^high^ patients treated with anti-VEGF agents received preoperative bevacizumab according to surgeon preference. CD31^low^ patients treated with anti-VEGF agents received aflibercept according to surgeon preference. DEGs were visualized via the heatmap.2 function in the gplots package in R. GSEA (v4.3.2) was performed via hallmark gene set collection via 1000 permutations.^86,87^

### The molecular signature of PDR monocytes

The molecular signature of PDR monocytes was established using relative expression order analysis (REOA).^88,89^ REOA is a rank-based approach that focuses on the relative expression ranking of genes and thereby enables comparison of transcriptomes generated from different tissue sources or sequencing methods. REOA was performed via the RankCompV3 package (v0.1.8) in R with the default settings.^90^ The molecular signature of PDR monocytes was established using REOA to compare the transcriptomes of PDR patient samples estimated to have >60% monocytes (patients L1 and L2) to those of retinal monocytes from a publicly available single-cell RNA-Seq dataset from the peripheral retinas of non-DM/DR human donors.^35^ The average monocyte expression was used for the non-DM/DR control transcriptomes. REOA was used to compare the relative expression ranking of each of the 17,430 genes expressed by either control monocytes (*n* = 3) or monocyte-enriched PDR samples (*n* = 2). DEGs were defined as those with an adjusted *p* < 0.05 (Data S1). Ingenuity Pathway Analysis was performed on the 289 differentially expressed genes that presented the greatest changes in expression (z1 > 100 or z1 < -100) in PDR monocytes.

### The molecular signature of PDR

The molecular signature of the PDR endothelium was established using REOA to compare the transcriptomes of endothelial cell-enriched PDR samples with those of endothelial cells from a publicly available scRNA-Seq dataset from the peripheral retinas of non-DM/DR human donors.^35^ Patients who received preoperative anti-VEGF therapy were excluded, and a separate cohort of three additional treatment-naïve endothelial cell-enriched samples was added to the four treatment-naïve CD31^high^ samples for this analysis. The three additional samples underwent the same cell and RNA isolation procedures, but were sequenced prior to the cohort described here. The average endothelial expression was used for the non-DM/DR control transcriptomes. REOA was used to compare the relative expression ranking of each of the 21,315 genes expressed by either control endothelial cells (*n* = 3) or the endothelial cell-enriched samples (*n* = 7). Differentially expressed genes were defined as those with an adjusted *p* < 0.05 (Data S3). Ingenuity Pathway Analysis was performed on the DEGs, excluding immunoglobulin genes. GSEA preranked hallmark gene set analysis was performed on the ranked list of genes generated by REOA via 1000 permutations.

### The molecular signature of OIR endothelial cells

The molecular signature of the OIR endothelium was established by performing REOA on P17 endothelial cells from a publicly available single-cell RNA-Seq dataset from OIR retinas (GSE150703).^26^ The cells from all timepoints were clustered via Louvain clustering of the top 6000 most variable genes at a resolution of 0.25 in Seurat.^91^ This clustering resulted in 18 retinal cell clusters (Supplementary Fig. 4). The endothelial cluster was identified via the markers *PECAM1*, *CLDN5*, and *VWF*. This cluster contained 63 endothelial cells from normoxic retinas and 60 from OIR retinas at P17. The transcriptomes of these endothelial cells were compared via REOA, which generated the molecular signature of OIR (Data S4). Differentially expressed genes were defined as those with an adjusted *p* < 0.05. The signature consisted of 770 DEGs, with 598 genes upregulated and 172 genes downregulated in the OIR endothelium.

### Statistics

All the statistical analyses were performed via R Studio. EdgeR was used for differential statistics in paired anti-VEGF comparisons, and the Benjamini‒Hochberg procedure was used to calculate the FDR. REOA was used to compare gene expression rankings in PDR and reference transcriptomes. This analysis determined DEGs in an unbiased fashion on the basis of the adjusted *p* value. All the graphs were generated via GraphPad Prism v9.5.1 (GraphPad Software). The data are presented as the means ± SDs.

### Study approval

The inclusion criteria were the presence of PDR and the need for vitrectomy surgery. All patients meeting these inclusion criteria were considered eligible for the study. The Institutional Review Board of the University of Illinois Chicago reviewed and approved the study design, under study ID STUDY2017-1176. Informed consent was obtained prior to inclusion in the study. Informed consent was also obtained for the use of fundus images. All procedures were in accordance with the Declaration of Helsinki for research involving human subjects. Sample size was determined by the availability of samples that yielded high-quality RNA-Seq data over the five-year study period. One outlier sample was removed from all analyses on the basis of a drastically reduced depth of sequencing.

## Supplementary information

Supplemental Material

Data S1

Data S2

Data S3

Data S4

## Data Availability

The gene expression signatures generated in this study are provided in the supplemental materials. The raw sequencing data and counts table have been deposited to the Gene Expression Omnibus database with the accession number GSE307925.
