# Supplementary material for: Proliferative diabetic retinopathy subtypes defined by immune defense and endothelial mitochondrial dysfunction
Source: Signal Transduct Target Ther. 2025 Oct 22;10:350. doi: 10.1038/s41392-025-02448-9 (PMC12541085; doi:10.1038/s41392-025-02448-9)
Supplement: Supplementary file 1 — Supplemental Material [file 41392_2025_2448_MOESM1_ESM.docx]

Supplementary Materials for

Proliferative diabetic retinopathy subtypes defined by immune defense and endothelial mitochondrial dysfunction

Maximilian A. McCann, Basma Baccouche, Yueru Li, Priti Roy, Neil Sheth, Jennifer I. Lim, William F. Mieler, Felix Y. Chau, Lawrence J. Ulanski, R.V. Paul Chan, Monique Munro, Robert A. Hyde, Caitlin Berek, Anna Ong, Sudeshna De, Barbara Siedlecki, Ru-Ik Chee, Yannek I. Leiderman, Michael J. Heiferman, Andrius Kazlauskas

Correspondence to: [ak20@uic.edu](mailto:ak20@uic.edu)

**This PDF file includes:**

Figures S1 to S4

Captions for Data S1 to S4

**Other Supplementary Materials for this manuscript include the following:**

Data S1

Data S2

Data S3

Data S4


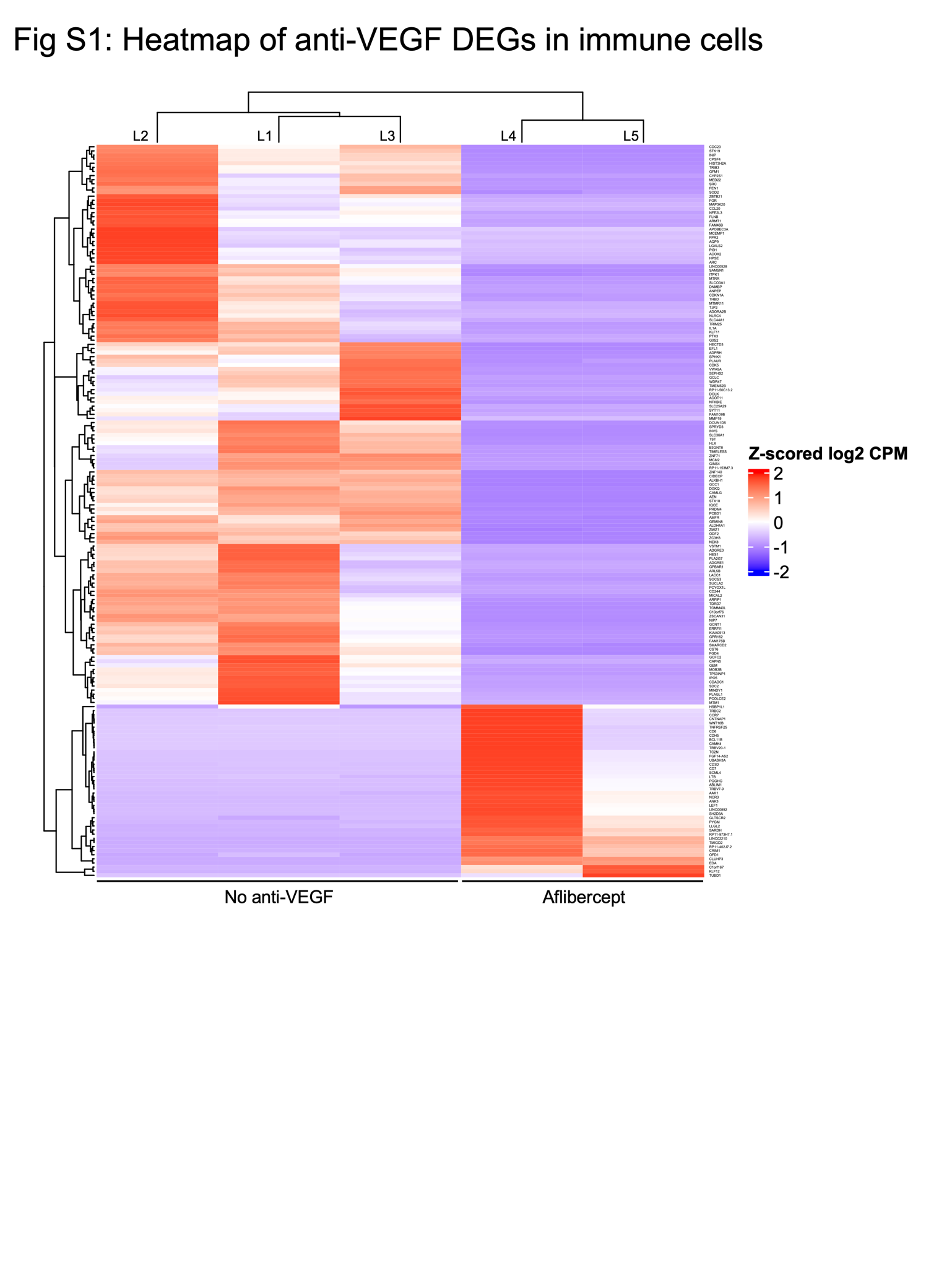


Figure. S1. Heatmap of anti-VEGF DEGs in immune cells.

Heatmap displaying the genes which are differentially expressed (FDR < 0.05) in the CD31^low^ populations from patients who received pre-operative aflibercept. Z-scores were calculated across all samples for each gene and mapped along a color gradient with negative z-scores in blue, zero in white, and positive z-scores in red. n= 2-3 patients per group. Gene names are also listed in Data S2.


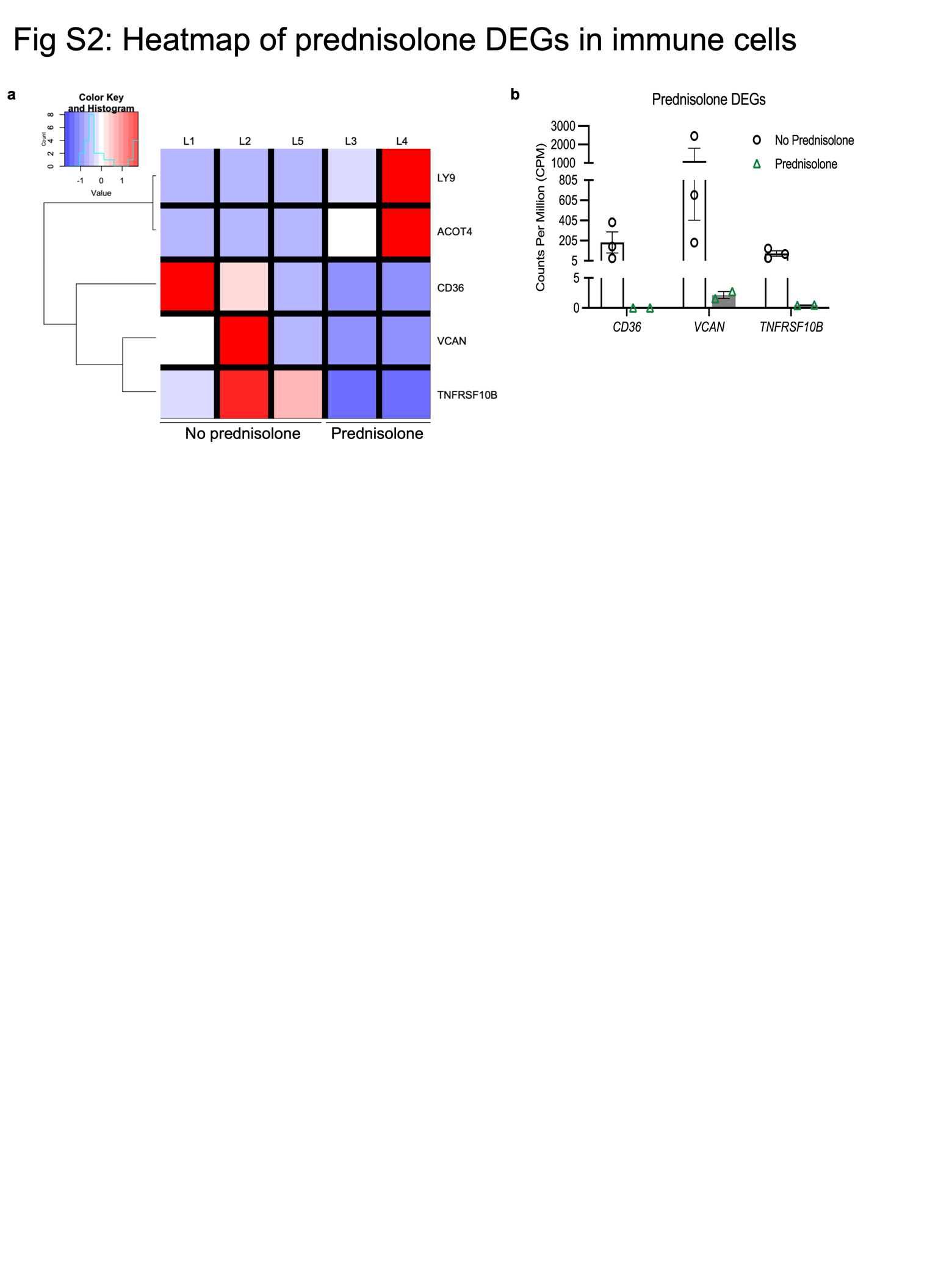


Figure. S2. Heatmap of prednisolone DEGs in immune cells.

**(a)** Heatmap displaying the genes which are differentially expressed (FDR < 0.05) in the cells from CD31^low^ patients who had systemic prednisolone in their medical records. Z-scores were calculated across all samples for each gene and mapped along a color gradient with negative z-scores in blue, zero in white, and positive z-scores in red. n= 2-3 patients per group. **(b)** Bar graph depicting the counts per million of *CD36*, *VCAN*, and *TNFRSF10B* in the cells from CD31^low^ patients who did and did not have systemic prednisolone in their medical records. Data are presented as mean ± SD, with each point representing a gene expression signature.


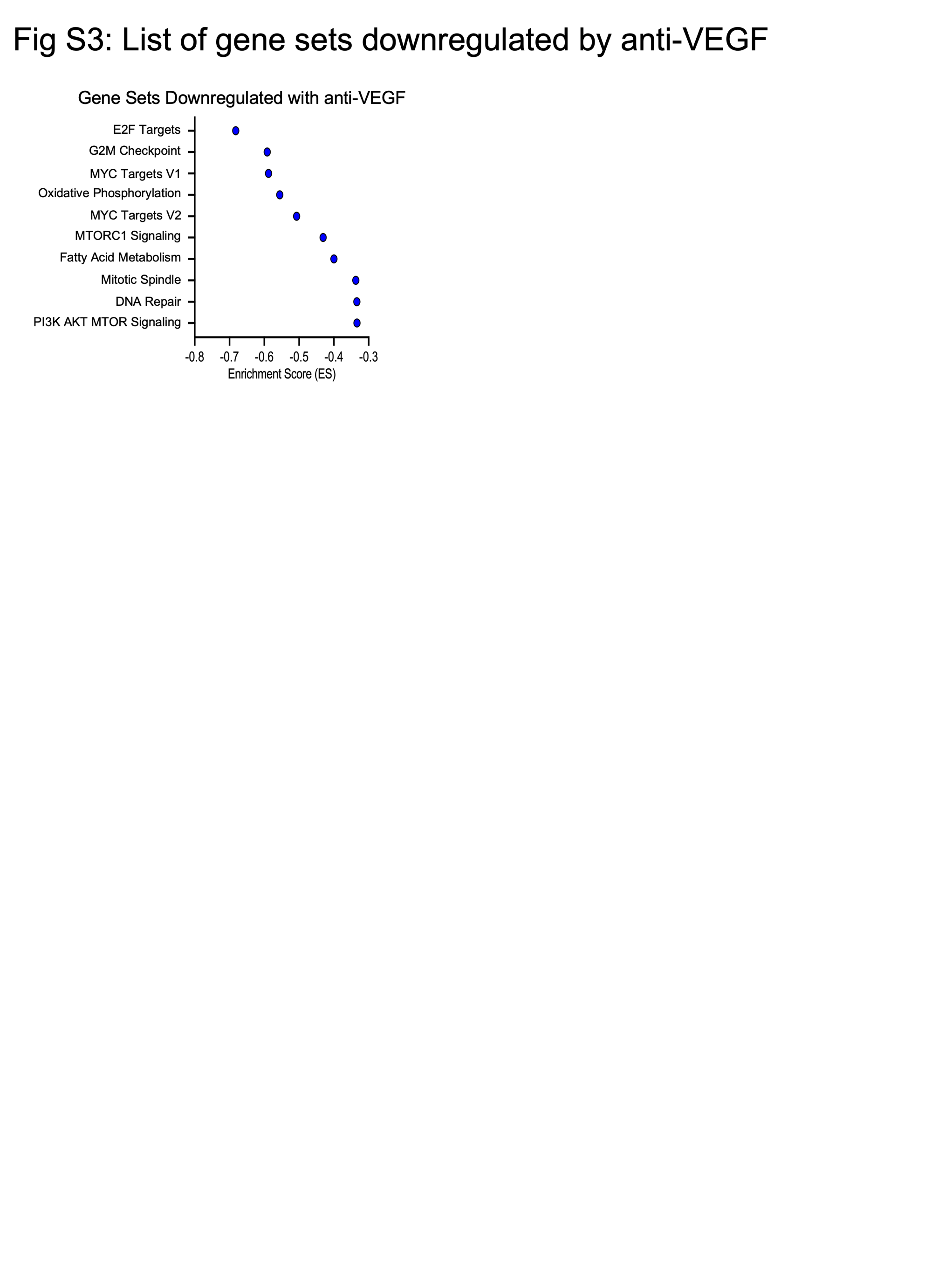


Figure. S3. Gene sets downregulated by anti-VEGF in EC-enriched samples.

Dot plots depicting the genes sets which were downregulated in the gene signature of CD31^high^ patients who received pre-operative anti-VEGF, as determined by Gene Set Enrichment Analysis (GSEA). Gene sets are organized by Enrichment Score.


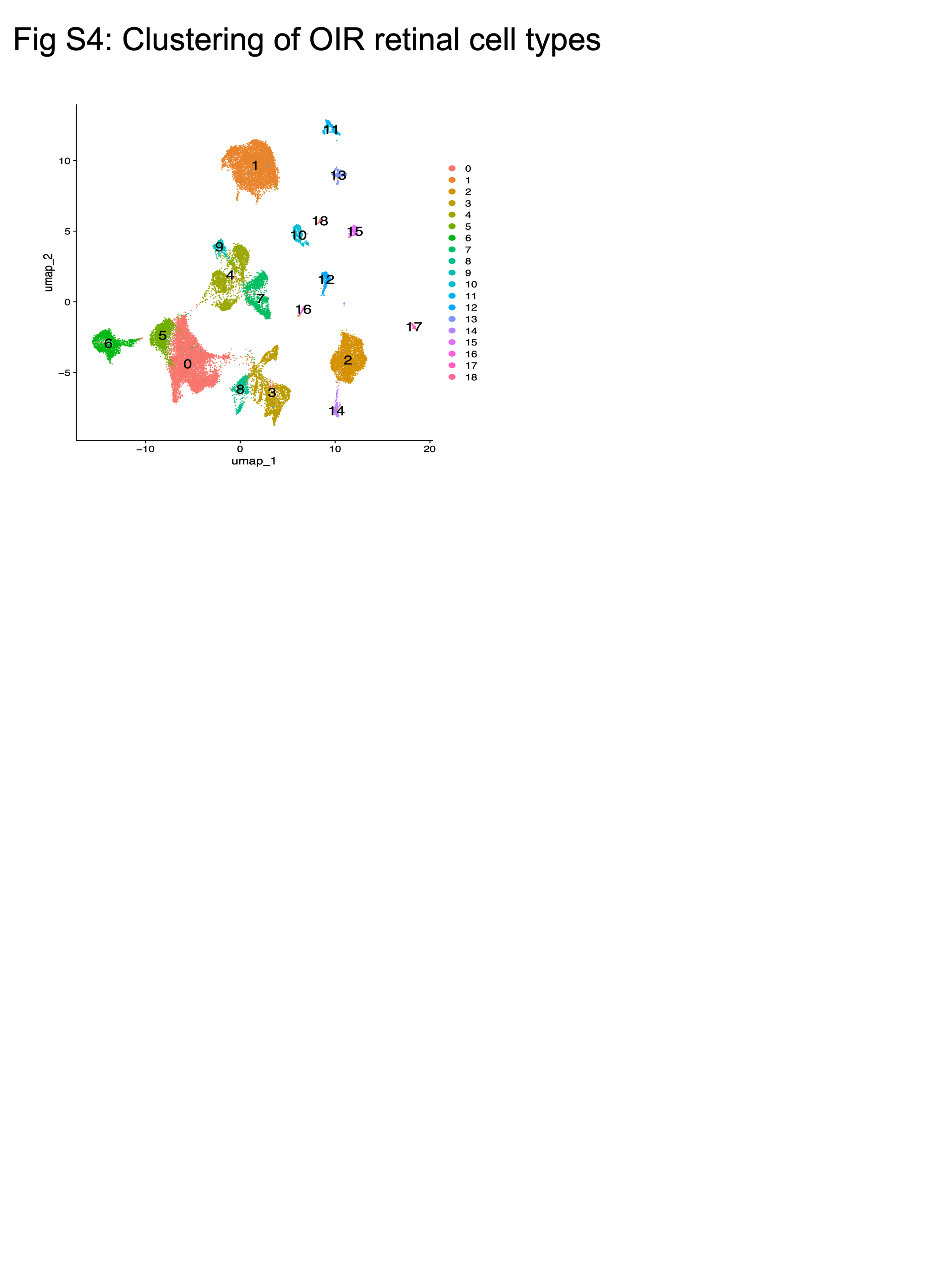


Figure. S4. Clustering of OIR retinal cell types.

Clustering of the cell types present in normoxic and OIR retinas from single-cell RNA-Seq dataset GSE150703.

**Data S1. Molecular signature of PDR in monocytes.**

List of differentially expressed genes (DEGs) in the monocytes of PDR patients. Genes are ordered by adjusted p value.

**Data S2. Anti-VEGF DEGs in CD31^low^ patients.**

List of anti-VEGF DEGs in the CD31^low^ samples. Genes are listed in the same order as the heatmap in Figure S1.

**Data S3. Molecular signature of the PDR endothelium.**

List of DEGs in the EC-enriched samples from PDR patients. Genes are ordered by adjusted p value.

**Data S4. Molecular signature of the OIR endothelium.**

List of DEGs in the endothelial cells from OIR retinas at P17. Genes are ordered by adjusted p value.
